# Potentiometric Response of Solid-State Sensors Based on Ferric Phosphate for Iron(III) Determination

**DOI:** 10.3390/s21051612

**Published:** 2021-02-25

**Authors:** Andrea Paut, Ante Prkić, Ivana Mitar, Perica Bošković, Dražan Jozić, Miće Jakić, Tina Vukušić

**Affiliations:** 1 Department of Analytical Chemistry, Faculty of Chemistry and Technology, University of Split, R. Boškovića 35, 21000 Split, Croatia; andrea.paut@ktf-split.hr; 2 Department of Chemistry, Faculty of Science, University of Split, R. Boškovića 33, 21000 Split, Croatia; imitar@pmfst.hr (I.M.); pboskovic@pmfst.hr (P.B.); 3 Department of Inorganic Technology, Faculty of Chemistry and Technology, University of Split, R. Boškovića 35, 21000 Split, Croatia; drazan.jozic@ktf-split.hr; 4 Department of Organic Technology, Faculty of Chemistry and Technology, University of Split, R. Boškovića 35, 21000 Split, Croatia; mice.jakic@ktf-split.hr; 5 Faculty of Chemistry and Technology, University of Split, R. Boškovića 35, 21000 Split, Croatia; tinavukusic@gmail.com

**Keywords:** ion-selective electrode, potentiometry, iron(III) cations

## Abstract

A novel ion-selective electrode with membranes based on iron(III) phosphate and silver sulfide integrated into a completely new electrode body design has been developed for the determination of iron(III) cations. The best response characteristics with linear potential change were found in the iron(III) concentration range from 3.97 × 10^−5^ to 10^−2^ mol L^−1^. The detection limit was found to be 2.41 × 10^−5^ mol L^−1^ with a slope of −20.53 ± 0.63 and regression coefficient of 0.9925, while the quantification limit was 3.97 × 10^−5^ M. The potential change per concentration decade ranged from −13.59 ± 0.54 to −20.53 ± 1.56 for Electrode Body 1 (EB1) and from −17.28 ± 1.04 to −24 ± 1.87 for Electrode Body 2 (EB2), which is presented for the first time in this work. The prepared electrode has a long lifetime and the ability to detect changes in the concentration of iron cations within 20 s. Membrane M1 showed high recoveries in the determination of iron cations in iron(III) standard solutions (98.2–101.2%) as well as in two different pharmaceuticals (98.6–106.5%). This proves that this type of sensor is applicable in the determination of ferric cations in unknown samples, and the fact that all sensor parts are completely manufactured in our laboratory proves the simplicity of the method.

## 1. Introduction

Ion-selective electrodes (ISEs) represent a small electrochemical sensor that can determine ion activity in various sample matrices without pretreatment. Potentiometry stands out as a non-destructive electroanalytical method with the possibility of application in a wide range of sensor performances [1]. Although liquid-contact ion-selective membranes are well developed and commercially available in a number of embodiments, their impossibility of miniaturization, special storage requirements, the sensitivity of inner filings toward evaporation the possibility of volume changes, and delamination of the sensing membrane, make solid-state electrodes preferred ion-selective sensors [2,3,4,5]. The possibility of developing this type of sensor with the characteristics of high selectivity and sensitivity and with the simplicity of implementation, low cost, and durability makes them attractive for use in ion determination in biological, chemical, environmental, and industrial samples [6].

Numerous solid-state ion-selective membranes with different compositions and performances for the determination of metal ions are known. Since iron is a ubiquitous metal in various biological processes and has found numerous industrial applications in steel, water pipes, paints, plastics, and the medical industry, it was not only desirable but necessary to develop a suitable method for rapid, efficient, and cost-effective iron determination. Some of the human health disorders are closely related to the imbalance of iron concentration in the human body, such as iron deficiency causes anemia, while iron accumulation can lead to the occurrence of hemochromatosis diseases [7].

Iron(III) cations are often detected by atomic absorption spectroscopy [8,9], inductively coupled plasma mass spectrometry [10], and spectrophotometry [11,12]. Although the aforementioned techniques provide accurate results, they are less accessible to the general population due to the necessary pretreatment of samples and sophisticated, expensive equipment that requires a trained analyst to use.

Considering different types of potentiometric sensors, ferric cations are mostly determined with membranes based on conductive polymers [7,13,14,15,16,17,18,19,20,21,22,23], carbon materials electrodes [24,25], and to a small extent with those based on iron salts [26].

In this work, a new homemade solid-contact ion-selective membrane based on sparingly soluble ferric phosphate is presented for the determination of Fe^3+^ cations in acidic media. To obtain a membrane selective for a particular ion, it is necessary to have a compound in the membrane composition that forms a stable complex or a sparingly soluble salt with the analyte. That compound represents the active centers of the sensors, and in this research is the iron phosphate precipitated in our laboratory. The other two components are silver sulfide, which is the charge transmitter, and polytetrafluoroethylene (PTFE) as the carrier. In this work, not only a completely new membrane for iron determination was presented, but also a new electrode body with many improved properties compared to the electrode body presented in previous work [27].

## 2. Materials and Methods

The main components of the membrane, iron(III) phosphate and silver sulfide, were prepared in our laboratory by a precipitation technique, while the polytetrafluoroethylene used was commercially available. The solutions used for both, preparation and testing of the proposed membranes, were prepared in ultrapure water with a declared conductivity of 0.04 µS cm^−1^ (Millipore Simplicity, Billerica, MA, USA). Ferric phosphate powder was prepared by mixing 0.5 mol L^−1^ anhydrous ferric chloride with disodium hydrogen phosphate of the same concentration under acidic conditions. A white-yellow precipitate was formed, which was centrifuged and washed with ultrapure water. After centrifugation, the ferric phosphate was dried in a vacuum dryer at 150 °C. Silver sulfide was also prepared by precipitation technique using silver nitrate and sodium sulfide nonahydrate as reagents. The black precipitate obtained, was then filtered, washed with chloroform and ultrapure water, and dried in a conventional dryer at 60 °C for 2 h. Different ratios of the three membrane components in the 500 mg mixture were homogenized and pressed under 625 MPa for 2 h to form membranes of 10 mm diameter. The compositions of the membrane mixtures are chosen by sequentially increasing or decreasing each of the three major components to investigate their influence. In membranes M1–M5, it was possible to find out which is the smallest percentage of the active substance in the composition to obtain a membrane that is selective towards iron cations. In membranes M6–M10, the percentage of polytetrafluoroethylene was reduced to find the smallest amount necessary to ensure the mechanical compactness of the membrane. Membranes M11–M15 show the importance of the presence of silver sulfide and polytetrafluoroethylene. A similar approach was previously reported [4,5,27,28]. As described, 15 different membranes were prepared and their composition ratios are shown in Table 1.

The XRD analysis of three main membranes components: FePO_4_, Ag_2_S, and polytetrafluoroethylene were performed by using Malvern PANalytical Empyrean X-ray diffraction system with the following operating conditions: Cu-Kα, 45 kV voltage and 40 mA current, 0.1050° step size, counting time/step of 88.5 s and a scanning angle 2θ from 5 to 80°. The instrument uses a multicore optics iCore/dCore and detector PIXcel3D-Medipix3 1 × 1 detector. In the procedure of data treatment collected pattern was corrected for systematic errors (external Si standard). The qualitative interpretation of the XRD pattern was made by comparison with standards patterns contained in the database PDF2 (ICDD, PDF2 Released 2020) by using HighScore Plus. Quantitative analysis was made by using the Direct Derivative (DD) quantification method. [28]

The ion-selective membranes for the determination of ferric cations presented in this work were tested in two different embodiments of the electrode body. They differ in terms of electrode size, membrane mobility, and the amount of charge transferred.

**Figure 1:**
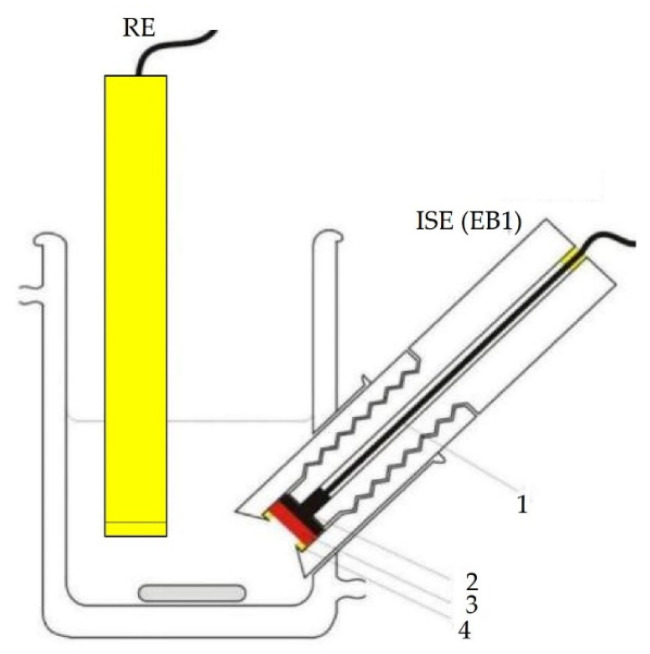
Scheme of ion-selective electrode (ISE(EB1)) and reference electrode (RE); (**1**—coaxial cable, **2**—steel disc, **3**—membrane, **4**—silicon rubber) [27].

**Figure 2:**
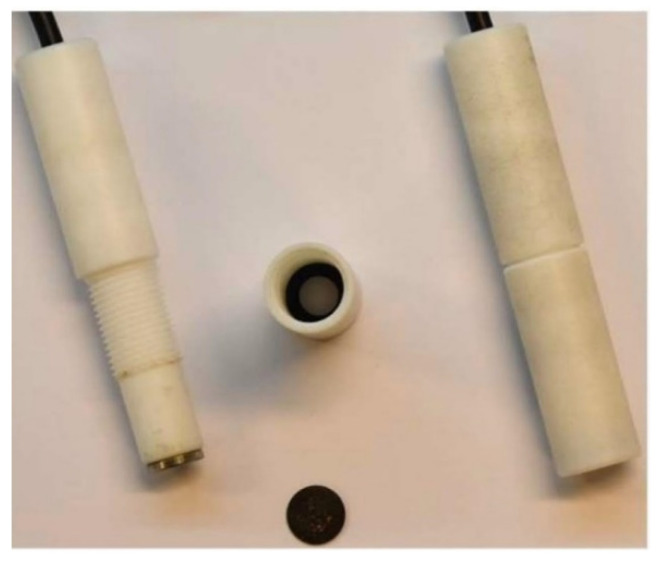
Digital picture of Electrode Body 1 (EB1).

The scheme of Electrode Body 1 (EB1) is shown in Figure 1 and the digital photo in Figure 2. It is entirely made of polytetrafluoroethylene, while the contact between the sensor and the millivoltmeter (SevenExcellence, Mettler Toledo, Switzerland-USA) is ensured by a stainless-steel disk connected to the device by a coaxial cable. The use of this electrode body requires a specially designed glass cell that ensures the positioning of the sensor at an angle of 45 degrees.

**Figure 3:**
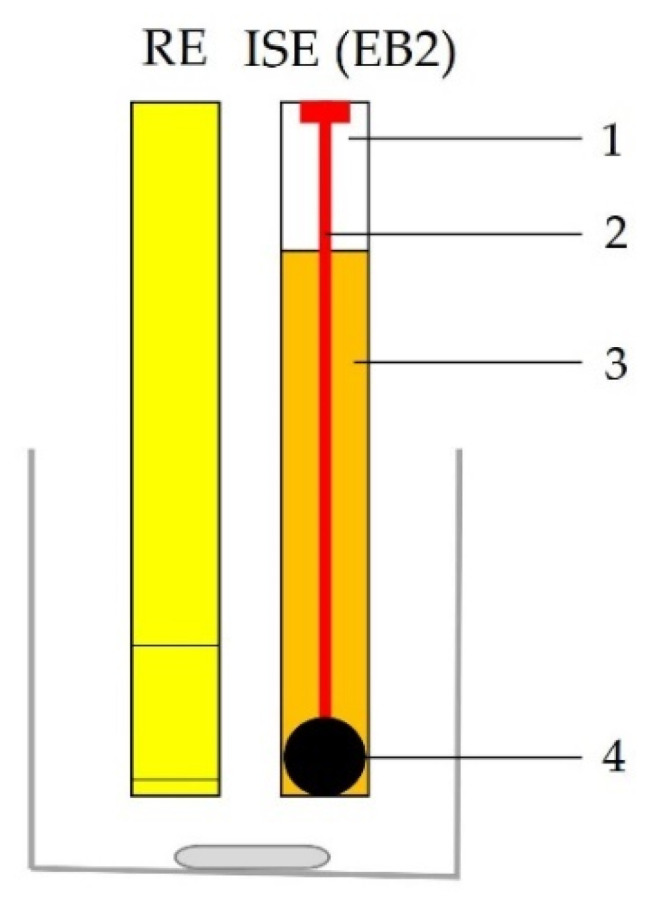
Scheme of ion-selective electrode (ISE(EB2)) and reference electrode (RE); (**1**—epoxy plate, **2**—copper layer, **3**—non-conductive lack, **4**—ion-selective membrane).

**Figure 4:**
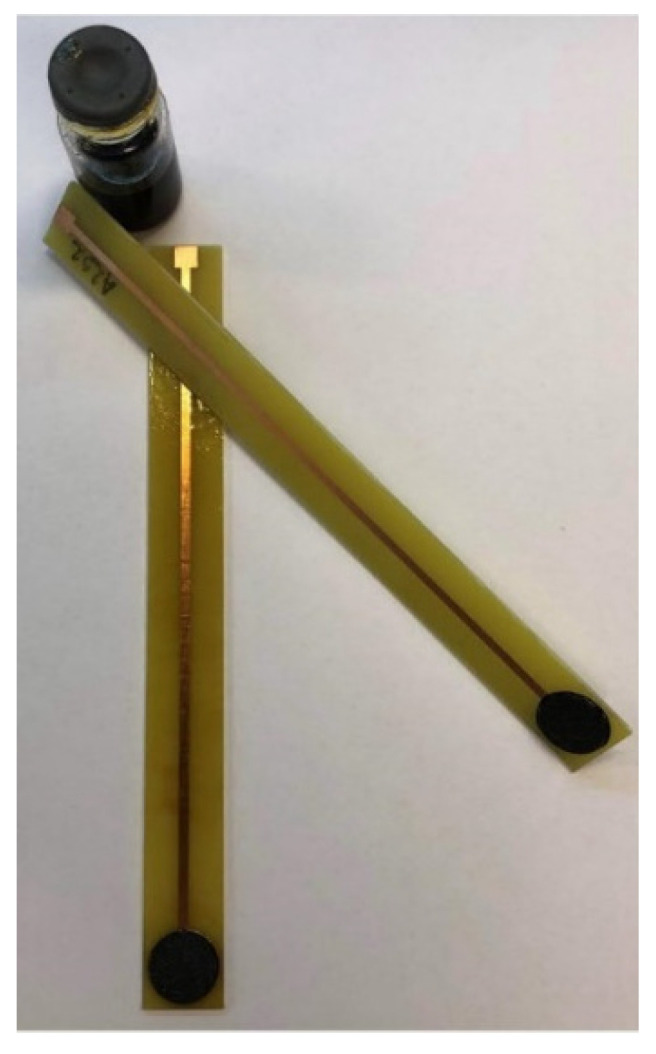
Digital photography of Electrode Body 2 (EB2).

The Electrode Body 2 (EB2), shown in Figure 3 and Figure 4 (digital photo), is based on the idea of print screen electrodes, it is miniaturized, not limited by the position angle and all tests can be performed in normal laboratory glass.

Although the idea of EB2 is based on the print screen electrodes, to the best of our knowledge, this type of design has not yet been reported. The copper layer on the epoxy plate provides charge transfer between the sensor and the cable connected to the millivoltmeter. Contact between the ion-selective membrane and the copper layer is made possible by a special conductive graphite adhesive. Since the adhesive is in a liquid state before drying, complete adhesion of the sensor to the tile is ensured and no loss of contact occurs. The copper layer is protected from the influence of the solution with a non-conductive layer in the form of a varnish commonly used to coat copper wires in cables.

A double silver-silver chloride electrode (Reference plus, Mettler Toledo) was used as the reference electrode in both cases. Both electrodes (reference and indicator electrodes) were immersed together in a solution containing ions of interest in a double-walled glass vessel (if EB1) or a conventional laboratory glass (if EB2) on a magnetic stirrer at room temperature.

Testing of all the presented membranes in Table 1 was carried out in anhydrous ferric chloride and ferric nitrate nonahydrate solutions at pH 0, 1, and 1.5 because at pH > 2 ferric hydroxide starts to precipitate which could prevent the membrane from responding. The pH of ferric chloride solutions was adjusted with sulfuric acid and ferric nitrate solutions with nitric acid. All sensors were also tested in the disodium hydrogen phosphate solution for the response towards phosphate ions at pH = 13, since under these conditions (PO_4_)^3−^ is the dominant form in a solution. The tests were carried out at room temperature by a standard dilution method.

Selectivity is one of the most important properties of an ion-selective electrode. It refers to the ability of the sensor to determine the presence of the analyte (A) over the presence of another (interfering) ion in the solution (B).

The matched potential method (MPM) was used to measure the selectivity coefficient since it was recommended in literature because of the different charge numbers of primary and interfering ions [29]. According to this method, the activity of Fe^3+^ cations was increased from *a*_A_ = 5 × 10^−4^ mol L^−1^ (reference solution) to *a*_A_’ = 5 × 10^−3^ mol L^−1^, and the potential change (ΔE) was recorded. In the next step, 0.1 mol L^−1^ solution of the interfering ion was added to the Fe^3+^ reference solution until the same potential change (ΔE) was recorded, and the concentration of added the interfering ion is thus *a*_B_. The values of the sensor selectivity coefficient are calculated using the following equation.

$$K_{A,B}^{\mathrm{pot}}=(a^{\prime }{}_{A}-a_{A})/a_{B}$$

The interfering species selected in this work were aluminum nitrate, barium nitrate, calcium nitrate, and magnesium nitrate. These interfering species were selected because of their high ability to form sparingly soluble precipitates with PO_4_^3−^ anions (Al(PO_4_), Ba_3_(PO_4_)_2_, Ca_3_(PO_4_)_2_), Mg_3_(PO_4_)_2_), according to their *K*sp values which are lower than 1 × 10^−20^.

To confirm the applicability of the sensor in the determination of ferric cations, a homemade sensor proposed in this work was used to determine Fe^3+^ ions in two different oxidized drugs. The dietary supplements used in this experiment were Tardyferon^®^ and Heferol^®^, both of which contain ferrous cations as active ingredients. Due to the complex composition of the supplements and the fact that it was necessary to oxidize ferrous cations to ferric cations, samples were prepared by microwave digestion using the Milestone flexiWAVE 480 (Milestone, Italy). One sample per insert was digested with nitric acid and hydrogen peroxide at a pressure of 0.75 MPa and a temperature of 180 °C in the vessel. The digestion process took 1 h. After cooling, a certain volume of the samples was diluted separately in two 100 mL flasks with nitric acid at pH 1.

## 3. Results

### 3.1. XRD Characterization

**Figure 5:**
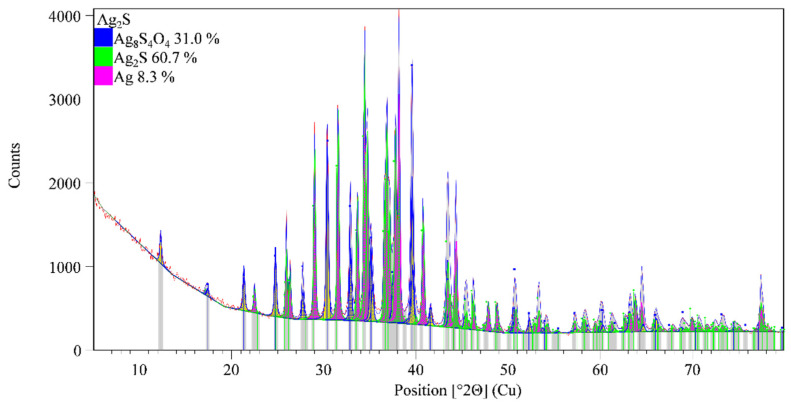
XRD pattern of silver sulfide.

**Figure 6:**
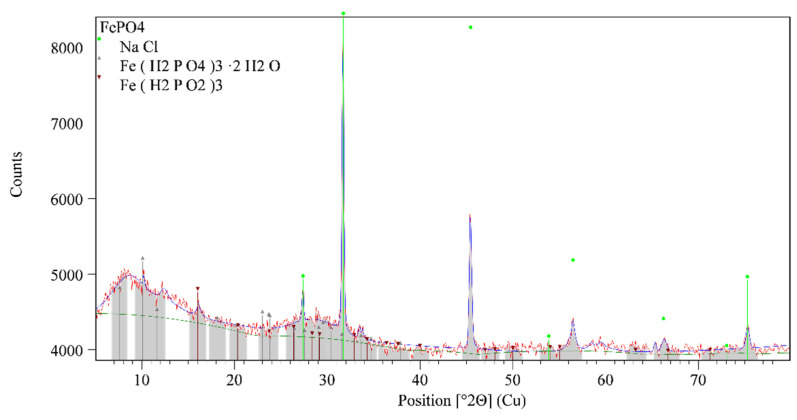
XRD pattern of ferric phosphate (FePO_4_).

**Figure 7:**
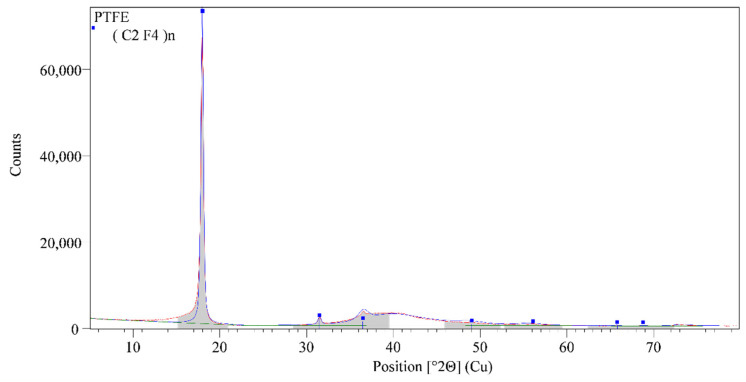
XRD pattern of polytetrafluoroethylene (PTFE).

Figure 5, Figure 6 and Figure 7 show the powder diffraction patterns of the prepared samples of silver sulfide (Ag_2_S), ferric phosphate, and used polytetrafluoroethylene for preparation membranes. According to the results of the analysis powder diffraction pattern of specimens denoted as Ag_2_S (Figure 5) is visible that its composition is a mixture of three different crystal phases, silver sulfide sulfate (PDF 00-061-0633), silver sulfide (PDF 00-068-0300), and silver (PDF 01-071-4613). The results of the conducted quantitative analysis of the diffraction pattern on the presence of phases silver sulfide sulfate, silver sulfide, and silver, indicate the proportion of phases is 31.0, 60.7, and 8.3 wt%, respectively.

The diffraction pattern of the sample denoted as FePO_4_ is shown in Figure 6, from the level of intensity background into diffraction patterns is visible high fluorescence which is presented into diffraction pattern which arises from the iron. The appearance of a diffuse diffraction maximum centered at 2Theta value at 8.72° and 29.08° indicates the presence of a structurally disordered phase (structurally ordered phase at the short-range distance). In addition, the sample also shows diffraction maxima that belong to the structurally arranged phases, according to the qualitative analysis diffraction pattern they belong to the NaCl (PDF 01-080-3939), Fe(H_2_PO_4_)_3_·2H_2_O (PDF 00-043-0104) and Fe(H_2_PO_2_)_3_ (PDF 00-001-0181). Figure 7 shows the diffraction pattern of the polytetrafluoroethylene used in the preparation procedure of membranes. Diffraction patterns show that PTFE is a semi-crystalline material that consists of the crystallized phase of polytetrafluoroethylene (PDF 00-061-1415) and the amorphous phase which suggests the appearing the diffuse diffraction maximum centered at 2Theta value at 39°.

### 3.2. Membrane Testing Results

**Figure 8:**
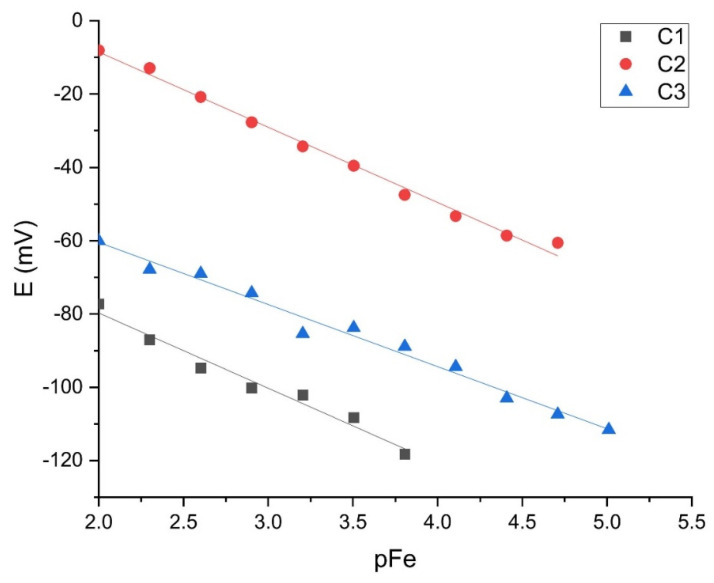
Results were obtained for Fe^3+^ cation determination in the ferric chloride solution using the M1 sensor.

**Figure 9:**
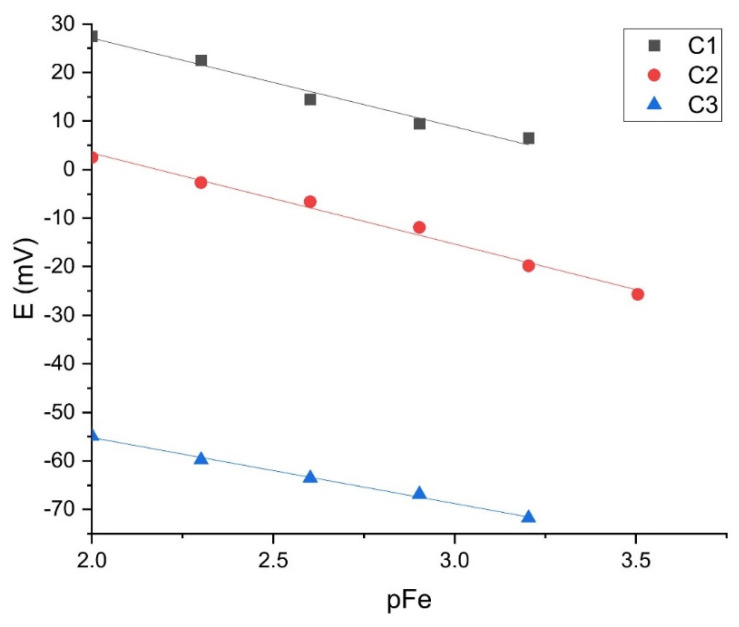
Results obtained for Fe^3+^ cation determination in ferric nitrate solution using the M1 sensor.

Among the numerous sources of ferric cations, membranes M1–M15 were tested in anhydrous ferric chloride, ferric nitrate nonahydrate, and disodium hydrogen phosphate solution. The main reason for choosing this kind of ferric solution was the possibility of chloride ions to form a sparingly soluble precipitate with the silver ions present in the phase boundary between membrane and solution while nitrate anions do not have this possibility. In this way, the response of the membrane to ferric cations was studied in the presence of interfering (Cl^−^) and non-interfering anions (NO_3_)^−^. The tests were performed with electrode bodies EB1 and EB2. In this way, all sensors were tested for response to ferric and phosphate ions. Membrane M1 with a composition of 25% FePO_4_, 25% Ag_2_S, and 50% PTFE, as it is shown in Table 2 exhibited a linear response to Fe^3+^ cations at pH = 1 with a potential change per decade in good agreement with the theoretical Nernst slope for trivalent cations in both, ferric chloride (Figure 8) and ferric nitrate solutions (Figure 9).

Table 3 shows the slope results of the M2–M15 test membranes in ferric cation solution at pH = 1. Since none of the M2–M15 membranes showed a slope close to −19.6 mV per decade, which would indicate the possibility of determining ferric cations, these membranes were not tested further.

Tests of the sensors for phosphate ions were performed at pH = 13 and none of the 15 membranes showed the same Nernst response towards (PO_4_)^3−^ as towards ferric cations at pH values of 0 and 1.5.

Although the membrane M1 tested in EB1 showed linearity in the range ([Fe^3+^] = 10^−2^–3.97 × 10^−5^) mol L^−1^ with a slope in good agreement with the theoretical Nernst slope for trivalent cations (−20.53 ± 0.63 mV per decade) and a high correlation factor (0.9925), there is no satisfactory repeatability among the measurements which could be caused by a periodic loss of contact between the sensor and the stainless-steel disc.

To ensure complete contact transfer, it was necessary to improve the adhesion of the sensor with the conductor, which is made possible by a special graphite adhesive in the EB2.

**Figure 10:**
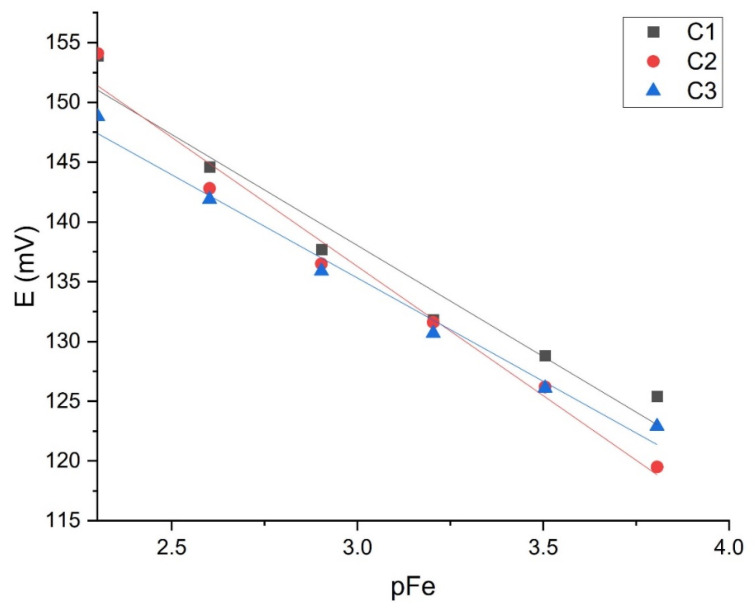
M1 intraday testing (*n* = 3).

**Figure 11:**
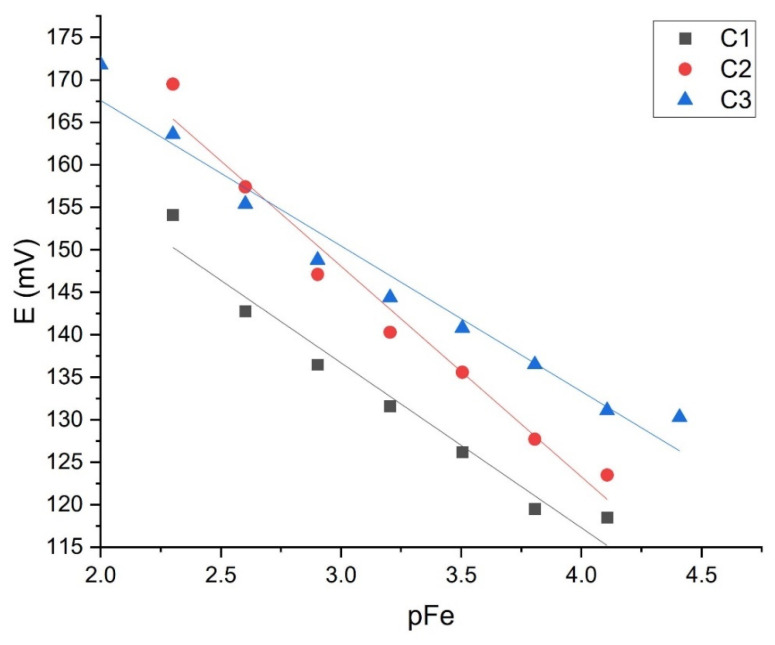
M1 interday testing (*n* = 3).

The sensor M1, which showed good results in the iron cation solution, was tested with EB2. Figure 10 and Figure 11 show the results of three tests intraday and three interday results. Table 4 shows slopes, LOD, and LOQ values with correlation factors of each curve (C1, C2, C3).

When comparing three consecutive measurements within one day, the graphical representation of the curves shows obvious high repeatability. The membrane showed a slope of −19.421 ± 1.70 mV per decade with a correlation factor of 0.9631. The detection limit is [Fe^3+^] = 1.43 × 10^−4^ mol L^−1^ and limit of quantification [Fe^3+^] = 5.86 × 10^−4^ mol L^−1^.

The M1 tests between days also show high repeatability of results with slopes from −18 ± 1.83 to −24 ± 1.87 mV per decade and correlation factor of from 0.9631 to 0.9761. The detection limit is [Fe^3+^] = (1.21 × 10^−4^–1.43 × 10^−4^) and limit of quantification [Fe^3+^] = (3.40 × 10^−4^–5.86 × 10^−4^) mol L^−1^.

From the graphs, it can be seen that the repeatability of the results is much better with EB2. As the results of the M1 membrane test showed the expected response, further experiments were carried out with this sensor only.

### 3.3. Electrode Selectivity

Table 5 shows the studied interferences with calculated selectivity coefficient values.

Table 5 shows that the values of the selectivity coefficients are very low, indicating high electrode selectivity to ferric cations. Although the presence of aluminum is the main interference for the determination of ferric cations, it is important to note that the concentration of Al^3+^ in a solution must be at least 50.4 times greater than the concentration of Fe^3+^ to cause interference (at [Fe^3+^] = 5 × 10^−4^ mol L^−1^).

### 3.4. Determination of Iron(III) in Pharmaceuticals

To confirm the applicability of the sensor M1 in the determination of ferric cations in the sample of unknown analyte concentration, it was tested in three different concentrations of Fe^3+^ standard solutions (Fe(NO_3_)_3_ × 9 H_2_O) at pH = 1. Recovery investigations of prepared membranes were done by using VWR 455532A iron standard for ICP and the results are shown in Table 6.

Since membrane M1 showed high recoveries (98.2–101.2%) in ferric standard solutions, the same sensor was used to determine Fe^3+^ ions in two different oxidized drugs. Samples of Tardyferon^®^ and Heferol^®^ prepared by microwave digestion described in detail above were analyzed potentiometrically for iron cations and the results were compared with those obtained by ultraviolet-visible spectrophotometric analysis. The spectrophotometric determination of iron cations was carried out by complexation with sulfosalicylic acid. Results are shown in Table 7 with calculated recoveries.

## 4. Discussion

The theoretical interpretation of the electrochemical behavior of precipitate-based ion-selective electrodes is based on the fundamentals of solubility equilibria and precipitation reactions at the phase boundaries of the electrode membrane. The fundamental reaction considered in this work is the reaction between phosphate ions (PO_4_)^3−^ present in the phase boundary between the membrane and the solution, and iron(III) cations present in the solution.

At the beginning of the design of sensors for iron cations, it was necessary to determine the appropriate composition of the three components of the membrane.

Fifteen different membranes were designed, each of which had a completely different composition. M1–M5 sensors were designed by gradually increasing the charge carrier ratio, M6–M10 sensors have the same ratio of FePO_4_ and Ag_2_S but a reduced ratio of PTFE. M11 and M12 sensors are without PTFE in their composition. M2–M11 sensors did not show suitable behavior for trivalent cations. The main reason for the unfavorable behavior of electrochemical potential is the absence of active centers on the membrane surface (Fe(PO_4_)_3_). Although polytetrafluoroethylene is an insulator and could be considered as interfering due to its inability to contact, the tests of M6–M10 membranes showed that reducing the amount of polytetrafluoroethylene did not improve the membrane behavior. Also, the tests of M11 and M12 resulted in rupture of the membrane and its one-time use, which is exactly the consequence of the absence of support. The M1 sensor is obviously the perfect combination of the set of active sites, charge transmitters, and carriers that provided a potential change as a function of the change in the activity of the iron cations in accordance with the requirements of the Nernst equation. Positive slope values recorded in a high percentage during the membrane test in ferric chloride solution could be due to the backreaction of the membrane to chloride anions. None of the sensors showed any response to phosphate anions, which is another indication that the M1 sensor is selective only for ferric cations.

After determining the ideal membrane composition, two variants of the electrode body were investigated, of which EB1 was presented in previous papers [4,5,27] and EB2 for the first time in this work. Both bodies were designed in our laboratory. Membrane tests in both bodies showed results in the style of the Nernst equation requirements, such that slopes in the range of −13.59 ± 0.54 to −24.78 ± 1.87 mV per decade were recorded. As explained in the Results section, the graphical representation of the M1 membrane test in the EB2 body shows an almost complete overlap of the three curves (C1, C2, C3) of three consecutive measurements, indicating high repeatability of the results within one day. The curves showing test results within three consecutive days show slightly larger deviations in the E_0_ value, but this is also to be expected. For this reason, when determining the concentration of iron cations in a standard or in real samples, the calibration curve must be prepared immediately before the measurement. The main disadvantage of the EB1 design is the non-uniform charge transfer. Namely, since both the membrane and the contact plate are in a solid-state and both have some non-uniformity at the microscopic level, in extreme cases they may touch each other only at one point, while in the other case they may adhere to each other completely. Since the silicone rubber that allows the membrane to be fixed inside the body under the influence of acids can be damaged, it often happens that the solution penetrates the space between the membrane and the stainless-steel disk, further preventing their contact resulting in none or Sub-Nernstian slope. The conductive graphite adhesive used in the construction of the EB2completely eliminates the aforementioned problem. Other advantages of the EB2 are: minimal saturation, increase in the working area of the sensor, the impossibility of replacing the working and adhesive sides of the membrane as it is solid, no need for special construction of the cell in which the electrodes are located, and general handling is much easier.

The iron ion-selective electrode (M1) described in this work with the possibility of application in two different electrode bodies (EB1 and EB2) showed a linear response towards iron cations in the range [Fe^3+^] = 3.97 × 10^−5^ mol L^−1^ − 1 × 10^−2^ mol L^−1^ with a change of −20.528 ± 0.63 mV per decade and a correlation factor of 0.9925. The sensor showed high selectivity to ferric cations in the presence of numerous interfering species and an average potential stabilization time of 20 s. The presence of aluminum cations caused the greatest interference to the determination of iron cations, which was expected according to the value of the solubility constant, *K*sp, of aluminum phosphate, which is 9.84 × 10^−21^. However, it is important to emphasize that as mentioned in the Results section, when the concentration of iron cations is 5 × 10^−4^ mol L^−1^, the concentration of aluminum cations causing interference must be 50.4 times higher. Other cations (Ba^2+^, Ca^2+^, Mg^2+^) do not cause significant interference and thus do not interfere with the determination of iron(III) cations. The M1 detection limit is [Fe^3+^] = 2.41 × 10^−5^ mol L^−1^ and the quantification limit is [Fe^3+^] = 3.97 × 10^−5^ mol L^−1^.

Due to the recoveries of Fe^3+^ determination in the standard solutions (98.2–101.2%), the M1 membrane was tested in real samples and the potentiometric results were compared with the UV/VIS spectrophotometry results. Recoveries of (98.6–106.5%) showed the reliability of using this homemade sensor for real sample analysis.

The time that has passed from the first to the last testing of the M1 membrane was around one year, so the conclusion is that lifetime of the sensor is from 10 to 12 months.

## 5. Conclusions

The ion-selective electrode, M1 showed a high ability to determine ferric cations in standard and pharmaceutical samples with recovery values ≈100%. Not only the possibility of using an M1 sensor in the real sample was reported but also high selectivity in the presence of many interfering species. This low-priced sensor showed high agreement with the requirements of the Nernst equation for trivalent cations. The potential change was –20.53 ± 0.63 mV per decade, with correlation factor *R*^2^ = 0.9925.

In contrast to many expensive methods for the determination of ferric cations in a sample, this method proved to be affordable and all sensor parts were manufactured entirely in our laboratory, demonstrating the possibility of wide availability.

Sensor M1 proposed in this work is suitable for qualitative and semi-quantitative analysis.

## Figures and Tables

**Table 1:** List of all tested membranes.

Sensor Name	Membrane Composition Ratio		
	FePO_4_	Ag_2_S	PTFE
M1	1	1	2
M2	1	2	3
M3	1	3	4
M4	1	4	5
M5	1	5	6
M6	1	4	3.33
M7	1	4	2.14
M8	1	4	1.25
M9	1	4	0.56
M10	1	4	0
M11	1	1	0
M12	2	1	0
M13	3	1	0
M14	1	0	0
M15	1	0	1

**Table 2:** M1 testing results in EB1.

Testing Solution	Linear Curve No.	Slope ± SD	LOD	LOQ	*R* ^2^
FeCl_3_ (pH = 1.00)	C1	−20.525 ± 1.56	2.64 × 10^−5^	8.97 × 10^−5^	0.9720
	C2	−20.528 ± 0.63	2.41 × 10^−5^	3.97 × 10^−5^	0.9925
	C3	−16.948 ± 0.72	1.31 × 10^−5^	2.63 × 10^−5^	0.9836
Fe(NO_3_)_3_ (pH = 1.00)	C1	−18.271 ± 1.57	1.13 × 10^−4^	4.52 × 10^−4^	0.9783
	C2	−18.755 ± 1.01	4.54 × 10^−4^	1.08 × 10^−3^	0.9885
	C3	−13.587 ± 0.54	1.55 × 10^−4^	8.20 × 10^−4^	0.9954

**Table 3:** Results of testing M2–M15 membranes in EB1 towards ferric cations.

Testing Solution	FeCl_3_ (pH = 1.00)	Fe(NO_3_)_3_ (pH = 1.00)		
Sensor	Slope/mV dec^−1^	*R* ^2^	Slope/mV dec^−1^	*R* ^2^
M2	11.49	0.9306	−4.95	0.7662
M3	3.09	0.3982	18.38	0.9176
M4	11.46	0.9900	17.47	0.4835
M5	24.68	0.7874	-	-
M6	11.27	0.9740	5.71	0.9881
M7	−4.42	0.0384	13.09	0.9496
M8	20.56	0.9813	-	-
M9	34.91	0.9193	1.69	0.8627
M10	22.00	0.9612	3.29	0.8586
M11	-	-	5.81	0.8828
M12	-	-	17.19	0.9744
M13	−9.01	0.9392	−27.74	0.8946
M14	–6.48	0.2376	-	-
M15	−6.91	0.9397	−11.02	0.9221

“-“—measurement could not be performed.

**Table 4:** M1 intra- and interday testing results in EB2.

Intraday Results					Interday Results				
Curve	Slope ± SD/ mV dec^−1^	*R* ^2^	LOD/ mol L^−1^	LOQ/ mol L^−1^	Curve	Slope ± SD/ mV dec^−1^	*R* ^2^	LOD/ mol L^−1^	LOQ/ mol L^−1^
C1	−18.58 ± 1.99	0.9563	3.27 × 10^−4^	1.83 × 10^−3^	C1 (day 1)	−18.53 ± 1.83	0.9761	1.21 × 10^−4^	3.40 × 10^−4^
C2	−19.421 ± 1.70	0.9631	1.43 × 10^−4^	5.86 × 10^−4^	C2 (day 2)	−24.78 ± 1.87	0.9724	1.31 × 10^−4^	4.43 × 10^−4^
C3	−17.28 ± 1.04	0.9855	2.38 × 10^−4^	6.31 × 10^−4^	C3 (day 3)	−19.421 ± 1.7	0.9631	1.43 × 10^−4^	5.86 × 10^−4^

**Table 5:** Logarithmic potentiometric selectivity coefficient values of M1 membrane in Electrode Body 2.

Interfering Species (B)	$\log K_{Fe^{3+},B}^{pot}$
Al^3+^	−0.74
Ba^2+^	−1.16
Ca^2+^	<−1.45
Mg^2+^	−0.82

**Table 6:** Determination of ferric cations in standard solution at pH = 1.

Added *m*(Fe^3+^)/mg	Determined *m*(Fe^3+^)/mg	Recovery (%)
0.84	0.85	101.2
1.68	1.65	98.2
8.37	8.28	98.9

**Table 7:** Determination of ferric cations in digested pharmaceuticals.

Pharmaceutical	Determined *m*(Fe^3+^) by M1/mg	Determined *m*(Fe^3+^) by UV/VIS/mg	Recovery (%)
Tardyferon	0.925	0.938	98.6
Heferol	1.283	1.205	106.5

## Data Availability

Not applicable.
